# Intracerebral Hemorrhage as the Initial Presentation of Chronic Myeloid Leukemia: A Case Report and Review of the Literature

**DOI:** 10.3389/fneur.2020.571576

**Published:** 2020-10-22

**Authors:** Huafeng Wang, Fei Cao, Jianhu Li, Ke Sun, Jie Jin, Ming Wang

**Affiliations:** ^1^Department of Hematology, College of Medicine, The First Affiliated Hospital, Zhejiang University, Hangzhou, China; ^2^Zhejiang Provincial Key Lab of Hematopoietic Malignancy, Zhejiang University, Hangzhou, China; ^3^Department of Neurosurgery, College of Medicine, The First Affiliated Hospital, Zhejiang University, Hangzhou, China; ^4^Bone Marrow Morphology Laboratory, College of Medicine, The First Affiliated Hospital, Zhejiang University, Hangzhou, China; ^5^Department of Pathology, College of Medicine, The First Affiliated Hospital, Zhejiang University, Hangzhou, China

**Keywords:** chronic myeloid leukemia, intracerebral hemorrhage, dasatinib, intracranial involvement, hyperleukocytosis

## Abstract

Intracerebral hemorrhage (ICH) is an unusual complication in chronic myeloid leukemia (CML). Intracranial involvement, causing ICH as an initial presentation is extremely rare in CML. Herein, we reported the first case of a newly diagnosed CML patient, who presented with headaches accompanied by nausea and vomiting as the initial presentations, caused by ICH. He underwent an emergency craniotomy twice and the postoperative pathologic examination confirmed intracranial CML involvement. Interestingly, his bone marrow and cerebrospinal fluid (CSF) smear and pathological study of the involved brain tissue showed proliferation of granulocytes, which were comprised mainly of metamyelocytes and myelocytes, without any blast within the brain tissue, suggesting the stage of CML was in the chronic phase (CP). He then received dasatinib treatment and achieved complete hematologic remission in the first 3-month follow-up but failed to reach a molecular response in the 6-month follow-up. By reporting this case and reviewing relevant references, we suggested intracranial CML involvement should be considered as a potential pathogenesis of ICH when the patient presents with hyperleukocytosis. A craniotomy is mainly for intracranial decompression and benefits the diagnosis of intracranial CML involvement. Tyrosine kinase inhibitors are effective in such patients to some extent, but more appropriate treatment strategies should be investigated in further detail.

## Introduction

Chronic myeloid leukemia (CML) is an asymptomatic disease. More than 30% of CML patients remain asymptomatic for a long period. CML can be classified into three phases: chronic phase (CP), accelerated phase, and blast crisis phase (BC). Intracerebral hemorrhage (ICH) is more common in acute myeloid leukemia, and CML of BC (1), than in CML of CP. Herein, we reported the first case of a newly diagnosed patient with CML, whose initial symptoms were headache accompanied by nausea and vomiting, caused by ICH with intracranial involvement at diagnosis.

## Case Presentation

**Figure 1:**
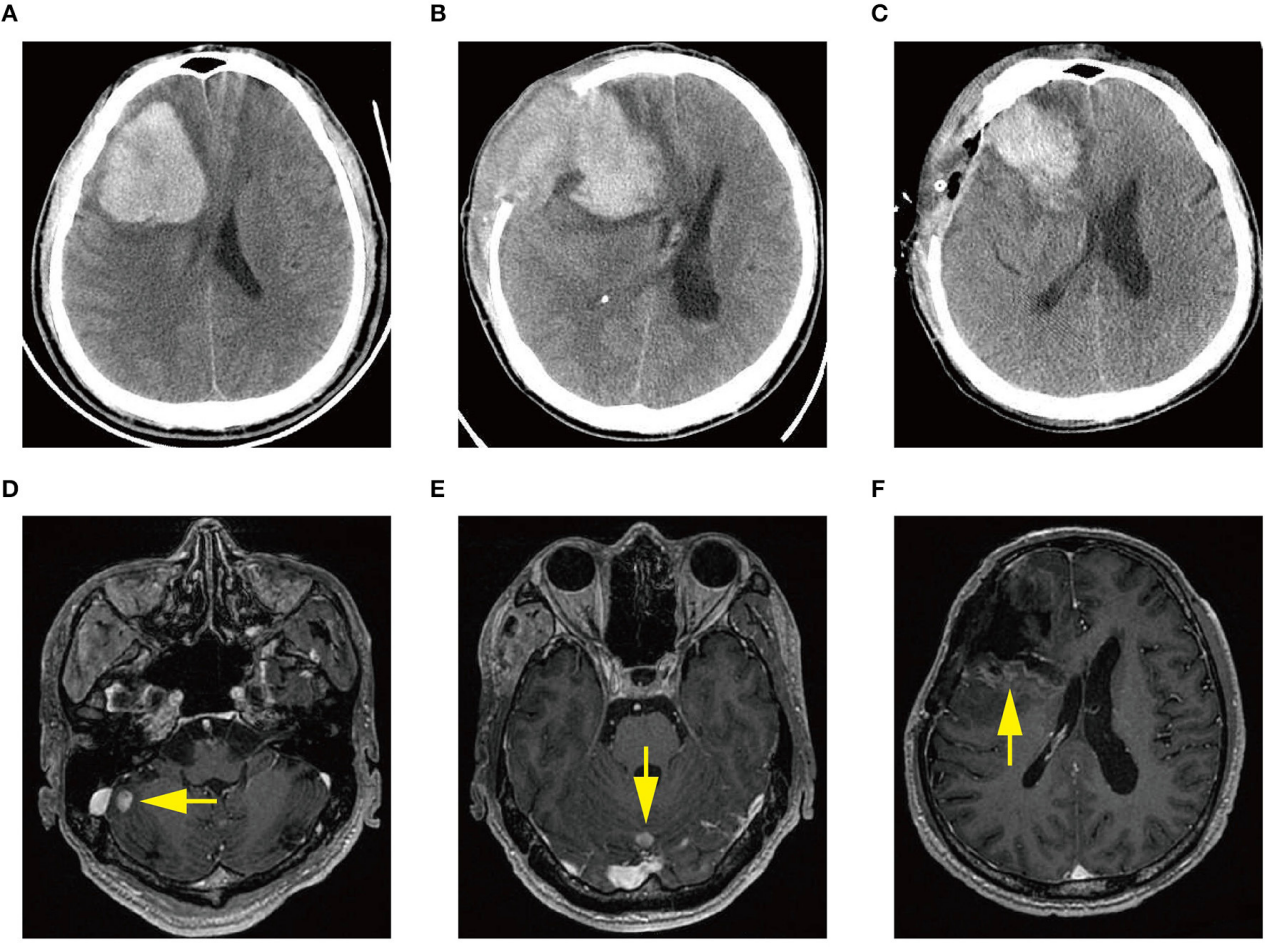
**(A)** Computed tomography (CT) scan at diagnosis demonstrating acute intracerebral hemorrhage (ICH) in the right frontal lobe. **(B)** CT scan demonstrating ICH re-occurrence at day 3 after the first craniotomy. **(C)** CT scan demonstrating rebleeding after the second craniotomy. **(D–F)** Magnetic resonance images showing multiple intracranial lesions (arrow) 1 month after the first craniotomy.

A previously healthy 45-year-old male was admitted to our hospital for a headache with nausea and vomiting for 2 days in November 2019. He presented without any risk factors for ICH, such as hypertension, diabetes, atrial fibrillation, and smoking or drinking history. On physical examination, we found his Glasgow coma scale (GCS) score was 13 (E3 V4 M6) (2). Pupils were bilaterally equal in size at 2 mm in diameter, with delayed light reflex. Muscular strength examination revealed the strength of muscle was grade 1 in the left limbs and grade 5 in the right limbs, respectively according to the manual muscle test grading system (3). Babinski's sign was positive on the left side. Computed tomography (CT) revealed a parenchymal hematoma about 74.8 ml in the right frontal lobe (according to the Tada formula), with peripheral cerebral edema and obvious middle-line shift (Figure 1A). A CT-angiogram did not find any cerebrovascular abnormality. Peripheral blood (PB) examination revealed leukocyte count of 571 × 10^9^ cell/L. The differential counts were neutrophils 44%, lymphocytes 2%, monocytes 3%, eosinophils 5%, basophils 3%, and abnormal leukocytes 43%. The hemoglobin level was 95 g/l and the platelet count was 218 × 10^9^/L. Abdominal ultrasonography showed splenomegaly. The initial impression of the patient was CML with ICH.

**Figure 2:**
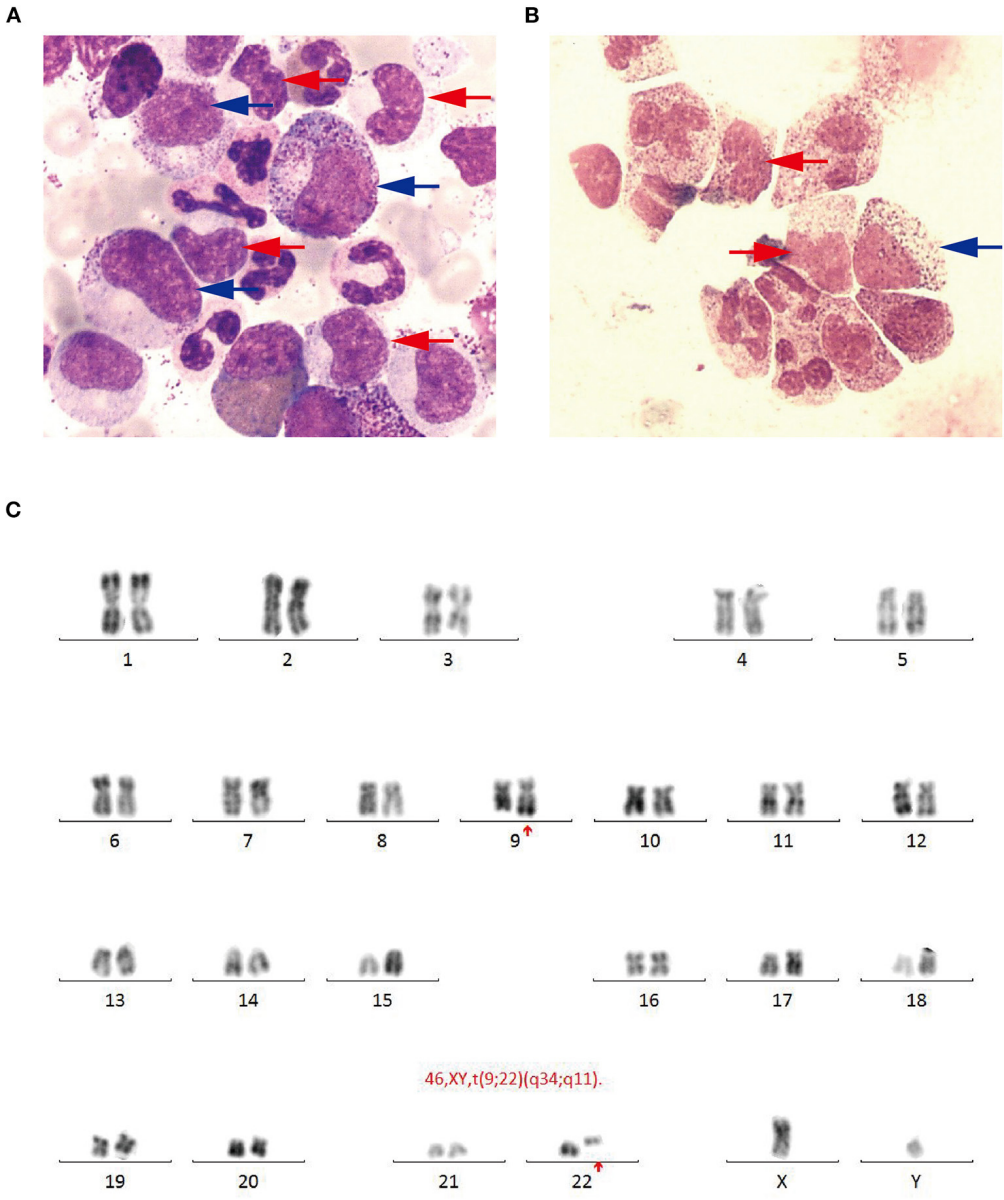
**(A)** Bone marrow smear (Wright-Giemsa, × 1,000) revealing active proliferation of granulocyte series, mainly metamyelocytes (blue arrow) and myelocytes (red arrow). **(B)** Cerebrospinal fluid smear (Wright-Giemsa, × 1,000) revealing abnormal granulocyte series, mainly metamyelocytes and (blue arrow) myelocytes (red arrow). **(C)** Chromosomal analysis revealing a Philadelphia translocation *t*(9;22)(q34;q11).

The patient received emergent craniotomy due to the marked mass effect caused by the hemorrhage. Intraoperatively, we found dark red tumor necrosis-like tissue and a green embolus-like substance in the hematoma. The cavity wall of the hematoma was composed of grayish-yellow abnormal tissue, which was richly supplied with blood. Following hematoma removal, the brain swelling persisted within the surgical area, so a decompressive craniectomy was performed. Post-operation, the patient was transferred to the intensive care unit, and he was stabilized, with normal blood pressure. He received mannitol 100 mg three times daily (q8h) for lowering intracranial pressure. His GSC score was 12 (E3 V4 M5), both pupils were equal in size; 2.5 mm in diameter, and the pathological sign was negative on day 1 after the operation. However, on day 3 after the operation, we observed unilateral dilation of his right pupil and the GSC score dropped to 9 (E2 V3 M4). Babinski's sign on the left was positive again, while repeated CT scans showed rebleeding in the surgical area with obvious middle-line shift (Figure 1B). Mannitol was increased to 100 mg four times daily (q6h), combined with furosemide 20 mg twice daily (q12h) to control intracranial hypertension. However, all these medical interventions did not work, and the consciousness level deteriorated with the GSC score dropping to 7 (E1 V2 M4). Therefore, a second craniotomy was performed. Once again, we evacuated the hematoma, tumor-like tissue surrounding the hematoma, and the blood-seeping temporal muscle. After the operation, his consciousness level improved. His GSC score became 13 (E4 V4 M5) and his right pupil restored back to its normal size. Muscular strength examination revealed the strength of muscle was grade 2 in the left upper limb, grade 1 in the left lower limb, grade 5 in the right upper limb and grade 4 in the right lower limb. The postoperative brain CT once more showed rebleeding in the operation area (Figure 1C). As this hematoma was much smaller than the previous one, the patient received conservative treatment. Hyperleukocytosis was considered as the possible pathogeny of ICH, so the patient was initially administered with hydroxyurea (1 g/day) immediately after his second craniotomy. Meanwhile, bone marrow (BM) aspirate and biopsy were performed to identify the diagnosis. The BM smear showed an active proliferation of granulocytes, mainly metamyelocytes and myelocytes (Figure 2A). Flow cytometry analysis demonstrated blast myeloid cells 1.72%, abnormal granulocytes 91.17%, and basophils 1.81% of non-erythroid cells. RT-qPCR showed the BCR/ABL P210 fusion gene at 69% positive. The chromosomal analysis identified a Philadelphia translocation t(9;22)(q34;q11) (Figure 2C), which confirmed the diagnosis of CML. As the presence of only 1.72% of blast cells in the bone marrow smear was detected, CML in CP was considered. Therefore, the patient was started on imatinib treatment (400 mg/day).

**Figure 3:**
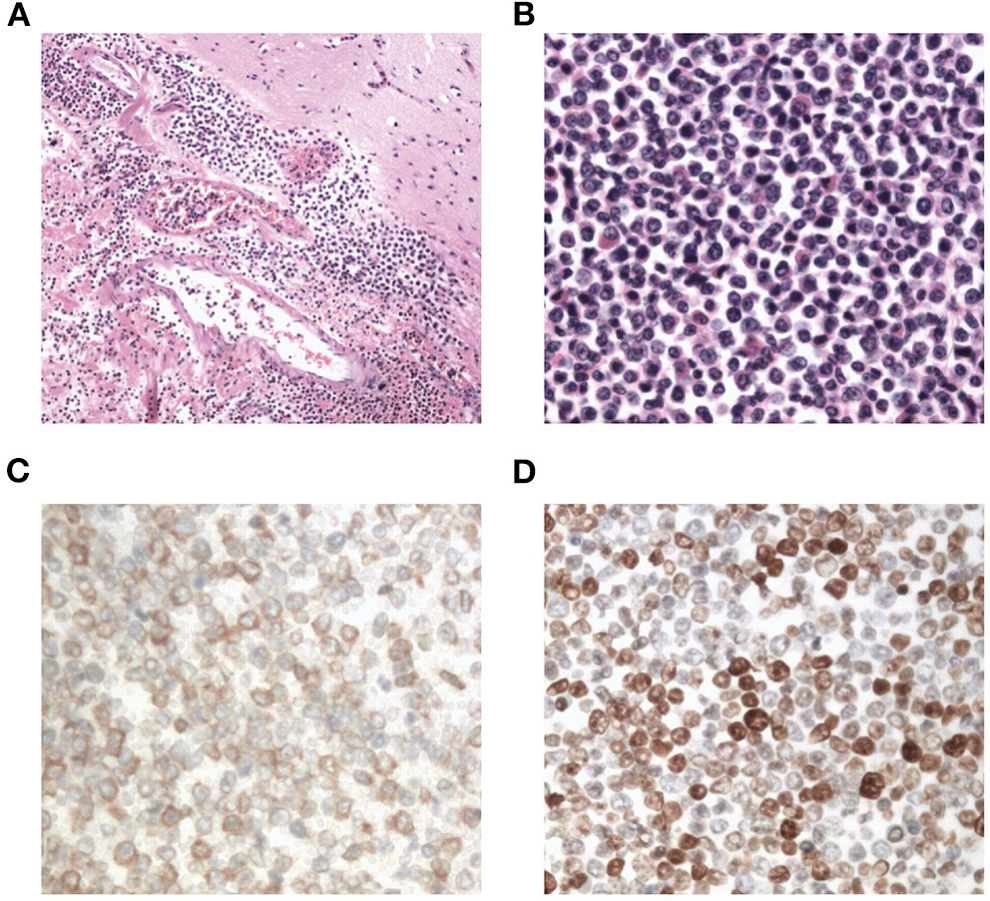
Photomicrographs of the resected brain tissue revealing myeloid cell hyperplasia with hemorrhage (hematoxylin and eosin stain **A**: × 100, **B**: × 400), and immunohistochemical staining demonstrating that tumor cells are positive for myeloperoxidase (**C**: × 400) and Ki-67(**D**: × 400).

Interestingly, the pathological examination of the brain tissue showed myeloid cell hyperplasia with hemorrhage in the tumor necrosis-like tissue. Immunohistochemical staining demonstrated that the cell was positive for MPO and Ki-67(30%), suggesting the diagnosis of intracranial involvement of immature myeloid cells (Figure 3). To further confirm it, a lumbar puncture (LP) was performed and xanthochromic cerebrospinal fluid (CSF) was found, with a red blood cell count of 4,950 cells/μL, nucleated cell count of 115 cells/μL, with neutrophils at 80%, lymphocytes at 6%, eosinophils at 4%, abnormal leukocytes at 10%, protein content of 0.69 g/L, and glucose content of 2.5 mmol/L. The CSF smear also revealed abnormal granulocytes, mainly metamyelocytes and myelocytes, without blast cells (Figure 2B). Flow cytometry of CSF showed abnormal granulocytes without blast, and FISH analysis confirmed the t(9;22) (q34;q11). Therefore, the patient received the diagnosis of CML with intracranial involvement. After 10-day treatment with imatinib, leukocyte count in PB returned to the normal level, but he still had a fever, postoperatively. Imatinib was then substituted with dasatinib (100 mg/day), for its better intracranial permeability, and because the fever was most probably caused by an uncontrolled intracranial CML. Three days later, his body temperature returned to normal. One month after operation, the patient's neurological function was recovered with a modified Rankin scale score of 2. The strength of muscle was grade 4 in the left limbs, and grade 5 in the right limbs. However, magnetic resonance (MR) showed multiple residual intracranial lesions, appearing enhanced on contrast-enhanced T1-weighted images (Figures 1D–F). The patient was asymptomatic, so he was discharged and asked to continue dasatinib treatment.

After being discharged from the hospital, the patient started neurorehabilitation. In the first 3-month follow-up, his modified Rankin scale score was improved to 1 and complete hematologic remission was achieved by February 2020. Although the BCR-ABL transcript level in PB was still 35%. A brain MR revealed the stable multiple intracranial CML lesions without a new hemorrhage and no other adverse events from dasatinib had occurred in the past 3 months. Although the genetic transcript level had not been effectively improved, the patient was satisfied with his current health condition. By simple oral administration, he achieved complete hematologic remission and agreed to continue dasatinib treatment.

**Figure 4:**
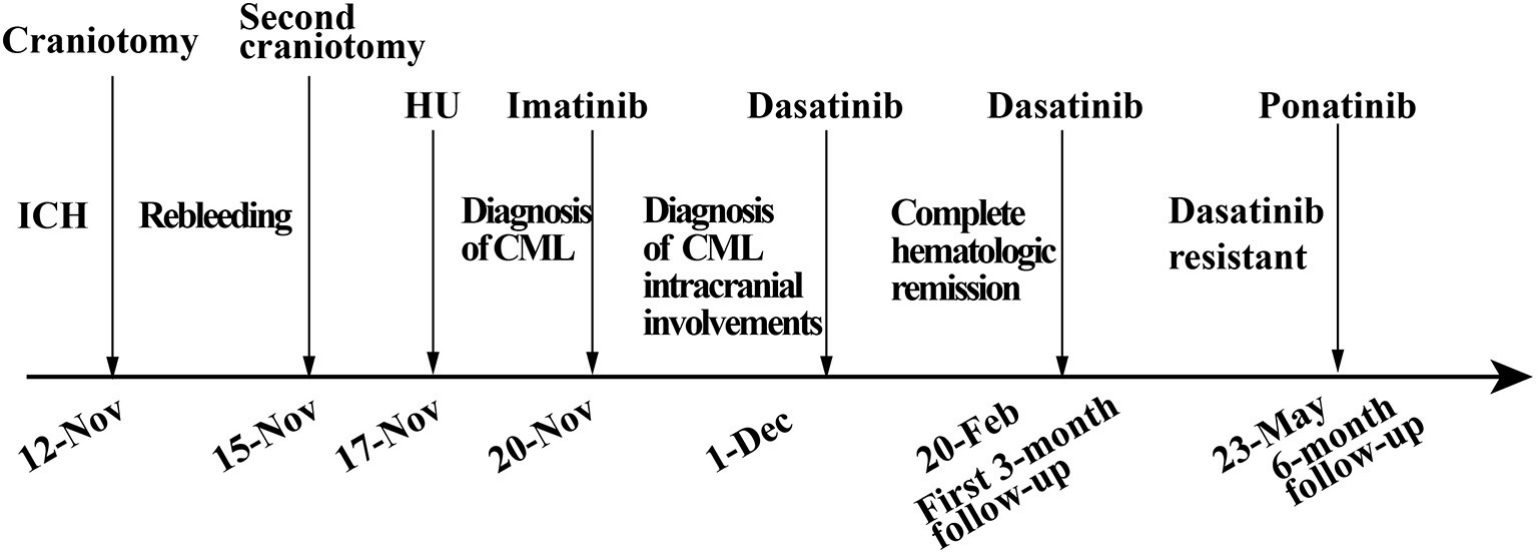
Timeline with relevant data from the episode of the case.

In the 6-month follow-up, the patient was able to engage in light manual labor with normal muscular strength in his limbs bilaterally. However, the BCR-ABL transcript level was still 14.9%, which suggested tyrosine kinase inhibitor (TKI)-resistant disease, according to the NCCN guidelines for CML (4). No mutation was found in the repeated bone marrow biopsy. Furthermore, the intracranial lesions which were enhanced at the 3-month follow-up had no enhancement by this time. Considering dasatinib resistance, we suggested the patient switch to third-generation TKI or receive additional chemotherapy. Due to concerns about the side effects of chemotherapy, the patient chose ponatinib, a third-generation TKI as his treatment. Overall, the patient was satisfied with the current treatment effect, but was still worried about the resistance of TKI (Figure 4).

## Discussion

Herein, we reported an extremely rare case of CML (CP? BC?) with intracranial involvement, presenting with ICH as the initial symptom. ICH is more common in acute myeloid leukemia or CML of BC (1) than in CML of CP. To the best of our knowledge, there are only seven cases of CML developing ICH in CP that have been reported so far (5–11) (Table 1). Among these cases, only one case presented with spontaneous ICH as the initial manifestation, but no intracranial involvement was reported. In light of the different criteria provided by IBMTR, MDADD, ELE, and WHO (12), the percentage of blasts in peripheral blood (PB) or BM should be more than 30 or 20% (per WHO) in CML of BC or there should be extramedullary blast proliferation (expect spleen) or large foci or clusters of blasts in the BM biopsy (per WHO). In our case, only 1.72% of blasts, mainly as metamyelocytes and myelocytes, were found in the BM smear, and no blast in the CSF or involved brain tissue, suggesting a diagnosis of CML in CP according to the above CML diagnostic criteria. However, multiple intracranial involvement sites and 30% Ki-67 positive cells in the pathological examination of intracranial lesions demonstrated that the tumor cells were highly proliferative and malignant, with less response to treatment. These characteristics made the diagnosis of CML in BC more likely. Therefore, multiple extramedullary CML involvement sites (except spleen), especially with high Ki-67(≥30%) suggested the diagnosis of CML in BC.

**Table 1:** Reported cases of CML with ICH in Chronic Phase.

**References**	**Age**	**Gender**	**WBC**	**PLT**	**PT**	**Initial manifestation**	**Location of hematoma**	**Treatment**	**Outcome**
	**(years)**		**(×10^**9**^/L)**	**(×10^**9**^/L)**	**(TNR)**				
Tsai et al. (6)	12	F	182	229	NA	Right hearing loss	R frontal and anterior thalamus	HU. IFNα,AraC, leukoapheresis	Dead (9d)
Muta et al. (7)	46	F	419	288	1.22	Retinal bleeding	Cerebellar	Leukoapheresis, AraC, imatinib	Alive
Cho et al. (8)	33	M	186	NA	NA	Hyperleukocytosis	R epidural,subdural and BG	Craniotomy, HU, imatinib, dasatinib	Alive
Kapur et al. (9)	52	M	685	164	1.6	Tinnitus and fullness in left ear	Multiple hemorrhage	HU,nilotinib	Alive
Aggarwal et al. (11)	60	M	37.1	350	NA	Headache	Intraventricular	Clipping	Dead (5d)
Olfa et al. (10)	26	M	260	291	NA	Headache after head trauma	R parietal lobe	No	Dead (12h)
Kouzuki et al. (5)	16	M	175	59	1.19	Fever and hyperleukocytosis	R temporal lobe	HU and then craniotomy	Dead (3d)
Present case	45	M	572	218	1.19	Headache, nausea, and vomiting	R frontal lobe	Craniotomy, HU, imatinib, dasatinib	Alive

*BG, basal ganglia; CML, chronic myeloid leukemia; F, female; HU, hydroxyurea; ICH, intracerebral hemorrhage; INR, international normalized ratio; M, male; NA, not available; PLT, platelet count at diagnosis; PT, prothrombin time; R, right; WBC, white blood cell count at diagnosis*.

The dysfunction of coagulation is the main etiology of ICH in CML of BC, while hyperleukocytosis may be the main pathogenesis of ICH in CML of CP (13). Hyperleukocytosis may result in leukostasis and vascular microthrombosis (14). Our case suggested that immature myeloid cells infiltrating the brain tissue was another possible pathogeny. This is the first case where obtaining brain tissue and CSF is essential to confirm intracranial involvement. Previously, Cho found epidural tumor evidence in a CML patient, but the pathogeny of ICH was not well-known (8).

Due to the extremely rapid deterioration and high mortality rate after the onset of ICH, half of the evaluable patients died within 10 days after administration, and they did not have a chance to get effective treatment for CML. Due to its rarity, the best treatment strategy for CML developing ICH remains controversial, especially for those cases presenting with ICH as the initial manifestation. An emergency brain CT is necessary for any patient presenting with symptoms suggestive of potential ICH and helps to determine the extent of the hematoma. Based on our case and previous cases (5, 8), we find it is easy to rebleed in CML with ICH. Therefore, a craniotomy is mainly for intracranial decompression and facilitates diagnosis. Hydroxyurea can be used before definitive diagnosis as a cytoreductive therapy, which is beneficial for reducing the risk of rebleeding. Once CML of CP is confirmed, TKIs are recommended, especially those with high intracranial permeability. Muta reported that imatinib was effective for their patient (6). Though in our case, imatinib did not work well, so it was replaced with dasatinib, a second-generation TKI with high intracranial permeability. Dasatinib effectively reduced abnormal granulocytes in CSF and controlled the body temperature, demonstrating its effect on the intracranial lesions. It is still necessary to stay vigilant to the risk of intracranial rebleeding, as dasatinib is occasionally associated with clinical bleeding among patients with CML, although CNS bleeding is relatively rare (15). Most of these hemorrhages are associated with severe thrombocytopenia. If this coincided with a normal platelet count, an additional factor might be involved in the pathogenesis of dasatinib-related ICH. Intracranial hypotension caused by LP was reported to be related to subdural hematoma associated with dasatinib (16). Our case achieved a complete hematologic response but a minor cytogenetic response after dasatinib treatment. If intracranial CML involvement is a diagnostic criterion for CML in BC, additional chemotherapy or a third-generation TKI such as ponatinib should be provided, according to the current BCR-ABL transcript level.

One important limitation in this case was a short duration of follow-up. Overall, the patient was satisfied with the current treatment effect. With oral administration, he can live and work as a healthy person. Although a longer follow-up is needed to evaluate the effect of ponatinib.

## Conclusion

In summary, we reported an extremely rare case of intracranial CML involvement with ICH as the initial presentation in a newly diagnosed CML patient. Intracranial involvement and hyperleukocytosis may be factors leading to ICH. A craniotomy is mainly undertaken for intracranial decompression and helps establish the diagnosis. Dasatinib is useful for controlling intracranial lesions, but it is necessary to stay aware of its side effect of bleeding. Further studies are needed to provide appropriate treatment strategies for these patients. More cases are needed to observe the overall survival and progression-free survival of CML patients with intracranial involvement.

## Data Availability Statement

All datasets generated for this study are included in the article/supplementary material.

## Ethics Statement

The studies involving human participants were reviewed and approved by Ethics committee of the First Affiliated Hospital, College of Medicine, Zhejiang University. The patients/participants provided their written informed consent to participate in this study. Written informed consent was obtained from the individual(s) for the publication of any potentially identifiable images or data included in this article.

## Author Contributions

HW was responsible for writing and the figure organization. MW and FC were responsible for the performance of operation. JL was responsible for collecting the bone marrow aspirate, biopsy, and examination. KS was responsible for the pathological examination. JJ was responsible for chemotherapy. MW was responsible for the FISH examination and manuscript review. All authors contributed to the article and approved the submitted version.

## Conflict of Interest

The authors declare that the research was conducted in the absence of any commercial or financial relationships that could be construed as a potential conflict of interest.
